# Assessing Familiarity and Interest in Dermatology Among Underrepresented Minority Pre-medical Students Following an Introductory Lecture

**DOI:** 10.7759/cureus.63785

**Published:** 2024-07-03

**Authors:** Fatuma-Ayaan B Rinderknecht, Semhar Teklu, Jenna Lester

**Affiliations:** 1 Dermatology, University of California San Francisco (UCSF) School of Medicine, San Francisco, USA; 2 Pathology and Laboratory Medicine, University of California San Francisco (UCSF) School of Medicine, San Francisco, USA

**Keywords:** inclusion, dermatology, skin of color, equity, diversity, medical education

## Abstract

Dermatology is the second least diverse specialty in medicine. This may be due in part to limited early exposure and the lack of familiarity among minority pre-medical and medical students. Our study evaluated an intervention where 62 pre-medical students attended a virtual dermatology seminar on May 6, 2022. The seminar introduced dermatology, highlighted key leaders of color, and provided an opportunity for questions and responses. Surveys assessing familiarity with and interest in dermatology were administered before and after the seminar. Data was stored in Qualtrics (Provo, UT) and analyzed using RStudio (Posit PBC, Boston, MA), with a response rate of 89% (n=55).

In the pre-survey, 20 students (32%) reported being familiar/very familiar with dermatology, compared to 47 students (85%) in the post-survey (P-value<0.001). Additionally, 26% (n=16) of students reported being likely to consider dermatology as a profession in the pre-survey versus the post-survey. These results suggest that targeted early-career interventions, such as this seminar, can increase familiarity and interest in dermatology among underrepresented in medicine (UIM) students, potentially contributing to greater diversity in the field.

## Introduction

Dermatology is the second least diverse medical specialty, with African American and Latinx doctors making up 3% and 4% of all dermatologists, respectively [1]. This limited diversity is due to multifaceted reasons, including limited familiarity and exposure to dermatology among minorities, financial barriers, the lack of mentorship and role models, and an emphasis on research and test scores in the application process [2-4]. A qualitative cross-sectional study found that underrepresented in medicine (UIM) dermatology applicants more often faced discouragement than non-UIM applicants [3]. They also reported a lack of group identity, noting the scarcity of role models who looked like them, and described the field as "overwhelmingly White" [3]. Applying to dermatology is costly due to the need to apply to many programs, research years, and away rotations, all of which pose financial barriers for UIM students or those from low-income backgrounds [2,3,5]. Additionally, the focus on the United States Medical Licensing Examination (USMLE) step scores and research has disadvantaged UIM students [2].

Research shows limited familiarity and exposure to dermatology among minorities. A 2016 study found that both privately and publicly insured African American and Latinx patients have fewer dermatology visits compared to their White counterparts [6]. A 2021 study found that African American college students had limited exposure to dermatology, with the most common source being social media [7]. Studies indicate limited early exposure to dermatology among UIM medical students [8], and of UIM medical students who matched to dermatology, 85% had participated in a pipeline program or had early exposure to dermatology [3].

Increasing diversity in dermatology is crucial not only for the workforce but also for reducing disparities in the field. Access to dermatology is limited in low-income, rural areas and counties with Native American, African American, or Latinx majorities [9]. Increasing minority physicians in dermatology could help ameliorate this problem as data shows that physicians from underserved backgrounds are more likely to serve underserved communities [10].

In this study, we created and administered an introductory dermatology seminar for a group of primarily UIM (African American, Latinx, Native American, or Pacific Islander) pre-medical students. Our primary objective was to assess the efficacy of a one-time intervention in increasing familiarity and interest in dermatology among UIM pre-medical students, which may aid in contributing to greater diversity in the field. The secondary objective was to gather data on which sections of the seminar most excited students and what future opportunities they found interesting.

## Materials and methods

Study design

This cross-sectional survey study aimed to evaluate the impact of a virtual introductory dermatology seminar on the familiarity and interest in dermatology among UIM pre-medical students.

Participants

The study involved 62 pre-medical students who attended a virtual one-hour introductory dermatology seminar on May 6, 2022. The participants were part of a mentorship organization known as the University of California San Francisco (UCSF)-University of California (UC) Berkeley White Coats for Black Lives, and the seminar was advertised to these students via social media platforms including Instagram and Facebook and an email Listserv. Convenience sampling was used due to the accessibility of this group. The eligible participants were 18 years or older, self-identified as pre-medical students, and completed more than 10% of the survey; all 62 participants were eligible.

Seminar structure

The seminar was led and created by a UCSF medical student (FR) and was reviewed for content and clarity by a UCSF dermatology faculty member (JL). The seminar was divided into four roughly equal parts. The first was an introduction to the field, which reviewed the path to becoming and the role of a dermatologist. This was followed by an interactive portion in which students identified skin diseases across various tones. Next, there was a discussion on disparities in the detection of skin conditions and a spotlight on hidradenitis suppurativa, which is an inflammatory condition disproportionately affecting African American populations [11]. The last portion highlighted minority leaders in dermatology and their work.

Survey structure

The participants completed an anonymous pre-survey immediately before the seminar and a post-survey directly after. The pre-survey and post-survey consisted of two questions assessing students' familiarity with and likelihood of pursuing dermatology as a career, using a five-point Likert scale from "not at all likely" to "very likely." The post-survey included 10 additional questions gathering demographic information and information on what portions of the seminar the participants enjoyed. Both surveys consisted of multiple-choice questions.

Study administration

The participants attended the seminar via Zoom™. Survey data was collected and stored in Qualtrics (Provo, UT). The surveys were administered online, with pre-surveys completed just before the seminar and post-surveys immediately after.

Data analysis

We utilized the test of equal or given proportions to compare the percentage of the participants who rated 4 or 5 on the Likert scale questions asking their familiarity with and likeliness to pursue dermatology in the pre-survey and post-survey. This analysis was conducted to determine if the differences between the pre- and post-survey responses were statistically significant, thereby assessing the effectiveness of the seminar in increasing familiarity with and interest in dermatology among the participants. Data was analyzed using RStudio (Posit PBC, Boston, MA), with a P-value of <0.05 indicating statistical significance.

Ethical considerations

This study was exempt from Institutional Review Board (IRB) approval by the Institutional Review Board of the University of California San Francisco (protocol number: 22-36377). Informed consent was obtained from all the participants before they completed the survey, ensuring voluntary participation and anonymity.

## Results

Participant characteristics

In total, 62 pre-medical students enrolled in the study; the response rate was 89% (n=55). The cohort was 74.6% (n=41) female, 23.6% (n=13) male, and 1.8% (n=1) gender non-conforming. Of the cohort, 47.3% (n=26) identified as Latinx/Latino and 20% (n=11) as African American, which was 2-4 times the percentage of African American and Latinx medical student applicants in the United States (8.7% and 9.5%, respectively) [12]. Most of the cohort was in the 18-25 age range (80%, n=44). Many participants came from disadvantaged backgrounds; 28.4% (n=38) identified as being of low socioeconomic status (e.g., recipient of Medicaid, Supplemental Nutrition Assistance Program {SNAP}, and Pell grants), and 27.6% (n=37) were the first in their family to graduate in college (Table 1). This was far above the proportion of US medical school matriculants from low socioeconomic or first-generation backgrounds, 6.0% and 10.8%, respectively [13,14].

**Table 1:** Participant characteristics SNAP: Supplemental Nutrition Assistance Program

	Number (%)
Gender	
Female	41 (74.6%)
Male	13 (23.6%)
Gender variant/non-conforming	1 (1.8%)
Race	
American Indian or Alaskan Native	0 (0%)
Asian	9 (16.4%)
African American or African	11 (20.0%)
Latinx, Latino, or of Spanish origin	26 (47.3%)
Middle Eastern/North African (MENA)	2 (3.6%)
Multiracial	4 (7.3%)
White	2 (3.6%)
Others	1 (1.8%)
Personal background	
Low socioeconomic status (e.g., recipient of Medicaid, SNAP, and Pell grants)	38 (28.4%)
First in family to graduate in college	37 (27.6%)
Grew up in an under-resourced rural or inner-city area	26 (19.4%)
First in family to attend professional/graduate school	20 (14.9%)
Refugee or first-generation immigrant	13 (9.7%)
Age	
18-22	32 (58.2%)
23-25	12 (21.8%)
26-30	8 (14.6%)
30+	3 (5.5%)

Exposure to dermatology

Of our 62 participants, 22 (40%) had visited a dermatologist, two (4%) had shadowed a dermatologist, and only one (2%) had participated in dermatology research (Table 1). Twenty students (32%) reported being familiar/very familiar with the field of dermatology in the pre-survey, whereas 47 students (85%) (P-value<0.001) reported the same after. Additionally, 26% (n=16) of our cohort said that they were likely to consider dermatology as a profession before the presentation, compared to 40% (n=22) (P-value=0.04) in the post-survey (Table 2).

**Table 2:** Differences between pre- and post-survey P-values were determined by utilizing the test of equal or given proportions; a P-value<0.05 is considered significant

	Number (%)	P-value
Familiarity with dermatology	Very familiar/familiar	
Pre-survey	20 (32.3%)	-
Post-survey	47 (85.5%)	<0.001
Likeliness to pursue dermatology	Very likely/likely	
Pre-survey	16 (25.8%)	-
Post-survey	22 (40.0%)	0.04

Participant areas of interest

**Figure 1:**
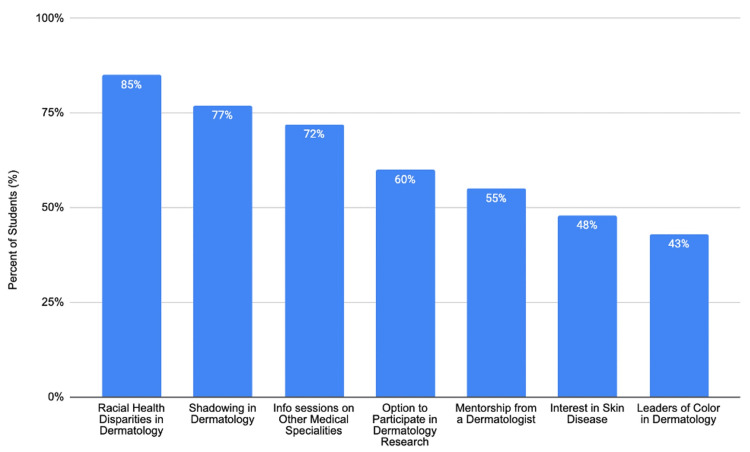
Subjects and opportunities in the seminar that interested the participants The bar graph shows the percentage of the participants interested in various seminar topics and opportunities: racial health disparities in dermatology (85%), shadowing in dermatology (77%), information sessions on other medical specialties (72%), dermatology research participation (60%), mentorship from a dermatologist (55%), interest in skin disease (48%), and leaders of color in dermatology (43%)

The participants were asked which aspects of the presentation piqued their interest; 85% (n=47) stated their interest in racial health disparities in dermatology, 77% (n=42) expressed interest in shadowing dermatologists, 72% (n=40) of the participants reported being interested in similar sessions on other medical specialties, and 60% (n=33) expressed interest in participating in dermatologic research (Figure 1).

## Discussion

Key findings and unique contributions

Our study demonstrated a significant increase in familiarity with and interest in dermatology among UIM pre-medical students following a one-hour virtual seminar. The percentage of students rating their familiarity with dermatology as 4 or 5 on a Likert scale increased from 32% to 85% (P-value<0.001), and those considering dermatology as a profession increased from 26% to 40% (P-value=0.04). These findings suggest that short-term, targeted interventions can effectively enhance awareness and interest in dermatology among underrepresented groups.

Our study also identifies that UIM pre-medical students have limited familiarity with the field of dermatology and that few had visited or shadowed a dermatologist. This finding is consistent with population research showing that minority, rural, and low-income communities have limited access to and exposure to dermatology [6,7,9]. Additionally, research has found that African American and Latinx medical students are more likely than non-UIM students to attend medical schools without dermatology programs, which also allows for less exposure to the field during medical school [15], further emphasizing the importance of interventions aimed at early exposure.

Novelty of the intervention

Previous research has established that UIM students often have limited exposure to dermatology [8]. However, interventions aimed at enhancing their interest in this field remain limited. While one existing study has demonstrated the efficacy of multi-session interventions with mentorship among minority high school students [16], our study is novel in that it finds that a shorter, more concise, educational intervention can also be effective in increasing interest in the field of dermatology among UIM pre-medical students.

Student interest in the skin of color dermatology

The participants expressed a strong interest and responded positively to learning about racial disparities in dermatology (85%) and hearing about minority leaders in the field (43%). Given that there are few UIM dermatologists and UIM dermatology faculty [1], it is likely that many pre-medical or medical students have not encountered other dermatologists with a shared racial identity. Previous studies have found that the lack of group identity is a barrier to UIM medical students who apply to dermatology [3]. This data suggests that highlighting UIM physicians and diverse patient populations may be an important part of interventions aimed at increasing UIM students' interest in the field.

Implications for future interventions

A significant proportion of the participants expressed interest in shadowing dermatologists (77%), participating in dermatologic research (60%), and receiving mentorship from dermatologists (55%). These findings indicate that mentorship, shadowing, and research programs are crucial for increasing diversity in dermatology, aligning with prior studies that show that such experiences enhance students' interest and perception of a medical specialty [17,18].

The lack of early exposure and research opportunities may pose barriers for UIM interested in dermatology. For example, matching into competitive specialties such as dermatology often requires several dermatology-specific publications and experiences; matched dermatology applicants had an average of 20.9 publications [5]. This may dissuade students who do not begin research early in their medical school careers from entering the field. Early exposure to a specialty in the first year of medical school has also been found to increase interest and consideration of the specialty as a career option [19,20]. Providing early exposure through seminars, mentorship, and research opportunities can be crucial in fostering interest and equipping UIM students to succeed in applying dermatology.

Lastly, given that 72% of our cohort expressed interest in similar sessions on other medical specialties, this intervention may serve as a blueprint for other fields with limited diversity, such as orthopedic surgery, plastic surgery, otolaryngology, and urology [1].

Limitations

This study is limited by its small sample size and the short-term nature of the intervention. Future research should include larger cohorts and longitudinal follow-ups to better understand the lasting impact of early exposure interventions on career choices in dermatology.

## Conclusions

In conclusion, this study introduced a novel educational intervention designed to expose and engage UIM students in dermatology. Our introductory seminar significantly enhanced the familiarity with and interest in the field among UIM pre-medical students, demonstrating the efficacy of our approach.

The seminar's focus on racial disparities in dermatology and highlighting leaders of color raised awareness about the unique challenges faced by minority groups and provided inspiration through representation. The participants expressed a strong desire for additional opportunities for mentorship, shadowing, and research, indicating that such resources could further support their interest in dermatology. The success of this intervention suggests that similar programs could benefit other medical specialties facing diversity challenges, making substantial progress toward a more inclusive and representative medical community.
